# Effects of adding electro-massage to manual therapy for the treatment of individuals with myofascial temporomandibular pain: a randomized controlled trial

**DOI:** 10.1590/1678-7757-2024-0109

**Published:** 2024-09-16

**Authors:** Luis ESPEJO-ANTÚNEZ, María de los Ángeles CARDERO-DURÁN, Alberto Marcos HEREDIA-RIZO, María Jesús CASUSO-HOLGADO, Manuel ALBORNOZ-CABELLO

**Affiliations:** 1 Universidad de Extremadura Facultad de Medicina y Ciencias de la Salud Departamento de Terapéutica Médico Quirúrgica Badajoz España Universidad de Extremadura, Facultad de Medicina y Ciencias de la Salud, Departamento de Terapéutica Médico Quirúrgica, Badajoz, España.; 2 Universidad de Sevilla Instituto de Biomedicina Departamento de Fisioterapia Sevilla España Universidad de Sevilla, Instituto de Biomedicina (IBiS) de Sevilla, Departamento de Fisioterapia, Sevilla, España; 3 Universidad de Sevilla Sevilla España Universidad de Sevilla, UMSS Research Group, Sevilla, España.; 4 Universidad de Sevilla Facultad de Enfermería, Fisioterapia y Podología Departamento de Fisioterapia Sevilla España Universidad de Sevilla, Facultad de Enfermería, Fisioterapia y Podología, Departamento de Fisioterapia, Sevilla, España.

**Keywords:** Temporomandibular disorders, Electrical stimulation, Manual therapies, Musculoskeletal pain, Pain assessment, Physical therapy

## Abstract

**Objective:**

To evaluate the effect of the addition of dynamic cervical electrical stimulation (electro-massage, ES) to manual therapy (MT), compared to MT by itself, in individuals with myofascial temporomandibular pain.

**Methodology:**

A total of 46 participants with bilateral myofascial temporomandibular pain for at least three months were distributed into two groups. Group 1 (n=21) received local MT consisting of soft tissue mobilization and release techniques over the neck and temporomandibular regions. Group 2 (n=25) received an ES procedure in the cervical region combined with the same intervention as group 1. All participants underwent a 2-week protocol. The primary outcomes were pain intensity (Visual Analogue Scale), pressure pain threshold (PPT) at the masseter and upper trapezius muscles (algometer), and pain-free vertical mouth opening (manual gauge). The secondary outcome was active cervical range-of-movement. Measurements were taken at baseline, immediately after intervention, and at a 4-week follow-up.

**Results:**

The ANOVA revealed significant changes over group*time, with better results for group 2 (large effect sizes) regarding pain intensity (p< 0.001; η2>0.14), pressure pain sensitivity and mouth opening (p<0.001; η2>0.14). Similar findings were observed for active cervical range-of-movement in all directions (p<0.001; η2>0.14), except rotation (p≥0.05).

**Conclusion:**

Electrical stimulation therapy over the cervical region combined with a MT protocol over the neck and temporomandibular joint shows better clinical benefits than MT by itself in subjects with myofascial temporomandibular pain. Registration code: NCT04098952

## Introduction

Temporomandibular disorders (TMD) are the second-most prevalent musculoskeletal disease that leads to pain and disability.^1^ It affects 10% to 15% of adults, mostly women,^2^ with an overall prevalence of 45% at ages 20 to 40 years.^3^ According to the International Classification of Orofacial Pain (ICOP), temporomandibular myofascial pain is the pain located in the masticatory musculature with or without functional impairment.^4,5^ It appears to be associated with cervical spine misalignment, neck pain, headaches, as well as stress, anxiety, and depression.^6-9^

The incidence of TMD and orofacial pain has increased steadily over the last years,^10^ with a global estimate of 34%, ranging from 26% in North America to 47% in South America.^11^ Patients with TMD often require a multimodal approach, not only with dentists and orofacial pain specialists, but also other health professionals specialized in the conservative management of orofacial pain associated with TMD^12^ through physical therapy, counselling, and relaxation techniques,^1^ among other procedures. Some physiotherapeutic interventions, such as manual therapy, stretching, coordination exercises, and electrical stimulation (ES) have been reported to be beneficial for people with myofascial temporomandibular pain.^3,13-15^ Manual therapy (MT) is the application of movement-oriented strategies integrating exercise and manually applied mobilization and/or manipulation techniques. For patients with TMD, MT usually includes mobilization or manipulation at the temporomandibular joint (TMJ) or cervical spine,^3,15,16^ as well as soft tissue techniques over the neck and masticatory muscles, although there is no clear consensus on the most effective approach.^17^ Transcutaneous electrical nerve stimulation (TENS) is the most investigated ES modality, with positive results for pain reduction,^13,18-20^ but with unclear results on the range of motion of TMJ or masticatory muscle activity.^18^ Interferential current electrical stimulation is another electrotherapeutic procedure that has also been shown to be effective in the treatment of musculoskeletal pain, usually in conjunction with other techniques.^21,22^ Dynamic ES delivered as an electro-massage has shown promising results in improving pain, function, and disability in adults with subacromial pain syndrome,^23^ and chronic low-back pain.^24^ This innovative way of application could improve pain and cervical range of motion in patients with TMD by modulating the autonomic response^24^ of the cervical spine muscles.

This study sought to assess the immediate and short-term effect (one-month follow-up) of adding dynamic ES (electro-massage) to a MT program, compared with the isolated used of MT, on pain-related measures, pain-free mouth opening, and cervical range of motion (ROM) in individuals with myofascial temporomandibular pain.

## Methodology

### Study design

The study was conducted as a controlled, randomized, single-blinded, parallel clinical trial, and complied with the Consolidated Standards of Reporting Trials (CONSORT) requirements. The research protocol was designed following the ethical, legal, and regulatory principals set in the Helsinki Declaration, and approved by the Ethical Research Committee of the Extremadura University, Spain (code 196/2019). The study has been registered on ClinicalTrials.gov, with code number NCT04098952.

### Participants

Following a convenience sampling, recruitment took place from November 2019 to October 2021 at a primary care rehabilitation center in Southern Spain, respecting the health recommendations to prevent SARS-CoV-2 infection. Individuals older than 18 years with a primary diagnosis of bilateral myofascial temporomandibular pain or diagnosis of primary myofascial orofacial pain, according to Axis I diagnostic criteria for TMD^25^ and the ICOP, respectively^4^ were included. Additional inclusion criteria were: (a) temporomandibular pain-related symptoms for more than three months before data collection; (b) current pain intensity at the masseter muscles over 3 cm on a Visual Analogue Scale; and (c) a score lower than 45 points on the Personal Psychological Apprehension Scale.^26^ The exclusion criteria were as follows: (a) previous surgery at the temporomandibular area; (b) current diagnosis of intraarticular damage (arthritis) or any other cause of inflammation at the TMJ; (c) a diagnosed of vestibular disorder; (d) having received any manual or physical therapy treatment in the previous two weeks; (e) or being under analgesic or anti-inflammatory pharmacological treatment. All participants provided a signed written informed consent before inclusion.

### Randomization and blinding

Randomization was performed using a computer-generated random sequence in permuted blocks. The sequence was obtained and safeguarded by a research assistant not involved in the trial. Sealed opaque envelopes, ensuring blind allocation, were prepared to conceal treatment order allocation into the two study groups. Evaluations and interventions were performed by two different therapists. The evaluator remained unaware of the participants’ allocation group.

### Interventions

All intervention procedures were conducted by the same physical therapist, who had over 15 years of experience in the clinical management of TMD. Patients assigned to group 1 carried out selected soft tissue techniques. Participants assigned to Dynamic ES (group 2) underwent the same program plus Dynamic ES (electro-massage). The study groups underwent a 2-week treatment regime (one session per week) at the primary care rehabilitation facilities. All the sessions were conducted on an individual basis. The interventions were implemented in accordance with the recommendations of the TIDIER statements.^27^ The Supplementary material shows the order of procedures for groups 1 and 2.

### Group 1: Manual therapy

**Figure 1:**
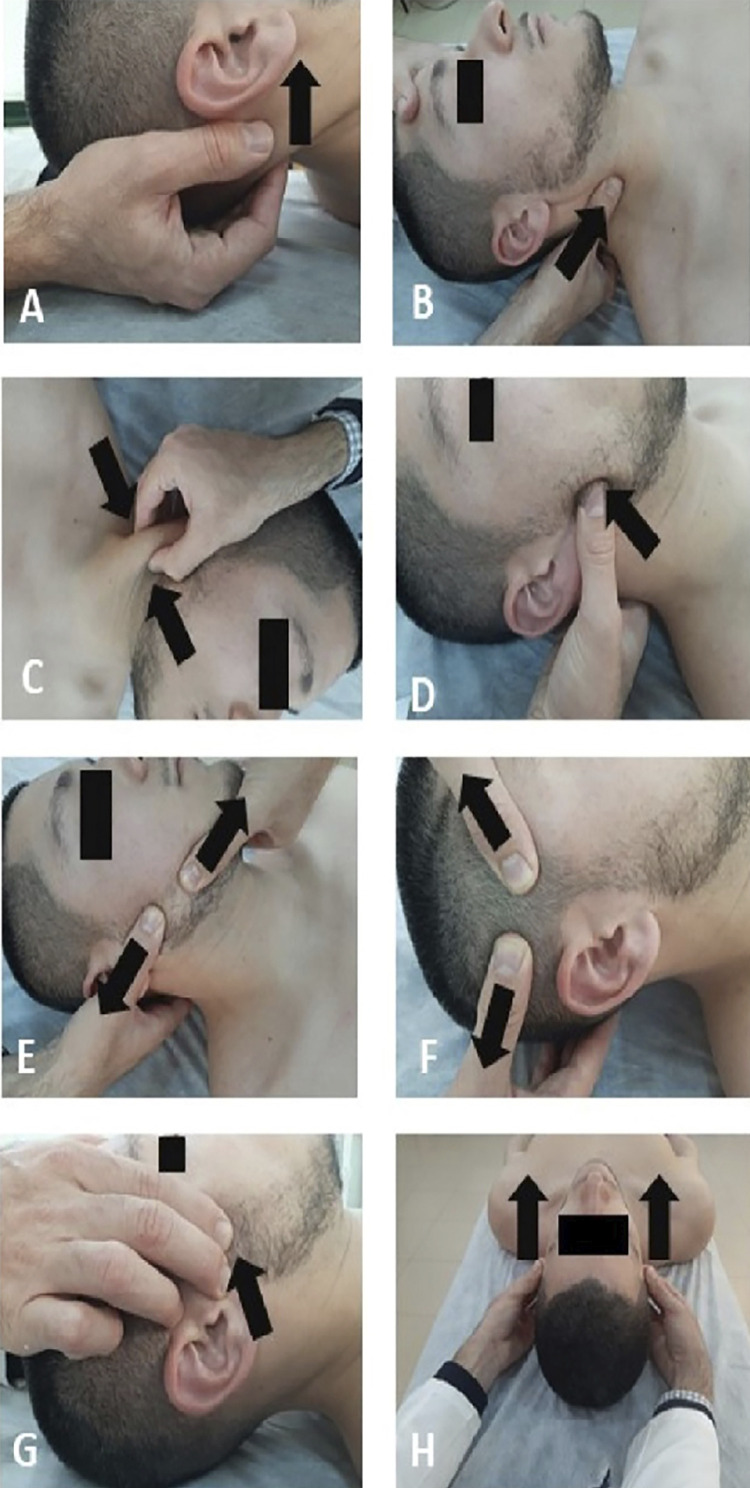
Cervico-temporomandibular manual therapy protocol. Suboccipital muscles inhibition technique (A); soft tissue mobilization of sternocleidomastoid (B and C), masseter (D and E), and temporalis (F) muscles. Decompression techniques at the temporomandibular joint (G and H).

Participants allocated to this group received a MT program consisting of soft tissue mobilization and release techniques over cervical and masticatory muscles. Previous randomized controlled trials have involved effective treatment protocols for patients with TMD, using both MT and exercise.^15,17^ Indeed, MT, as part of a multimodal conservative approach, continues to be recommended for the management of TMD.^28^ In our study, the different MT procedures included pressure release and inhibition techniques applied bilaterally over the suboccipital (Figure 1A), sternocleidomastoid (Figures 1B and 1C), masseter (Figure 1E) and temporalis (Figure 1F) muscles, with the patient in the supine position. All these techniques were conducted using a gentle pain-free pressure and repeated between three to five times. In addition, ischemic compression was used for the masseter muscles (90 seconds, two repetitions) (Figure 1D), and decompression techniques were applied to the TMJ (Figures 1G and 1H) (90 seconds, two repetitions). In total, the MT program consisted of eight techniques for a total of four muscles (suboccipital muscles, sternocleidomastoid, masseters and temporalis muscles). These muscles were chosen because their pain referral may be perceived around the TMJ, for the established overlap in nociceptive processing between cervical and trigeminal systems^29^ and for the relationship between the cranio-cervical region and the dynamics of the TMJ.^30^ The techniques were applied in the abovementioned order for all participants, as shown in figure 1. Finally, participants were advised that the manual pressure of the techniques may lead to pain, but tolerance was respected at all times. Each MT session lasted for approximately 25 minutes. The procedure is available in the supplementary material.

### Group 2: Dynamic ES (electro-massage) plus manual therapy

**Figure 2:**
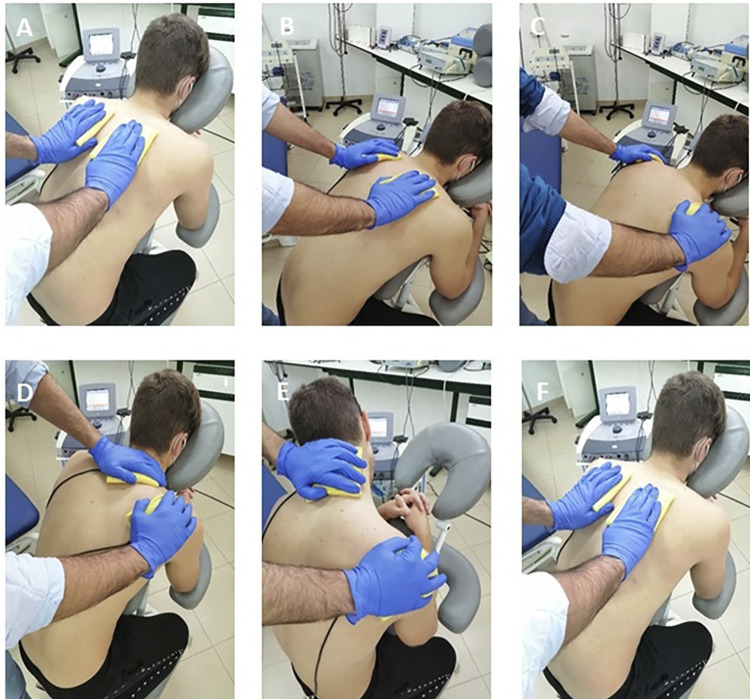
Interferential current therapy massage. Superficial sliding (A); deep sliding by itself (B) or combined with shoulder drop (C); transversal kneading over the trapezius (D); stretching of upper trapezius; and superficial sliding (F).

After the MT program, this group received a dynamic ES procedure (electro-massage) based on interferential currents (IFC) over the neck-shoulder region.^23,24^ The area of application was chosen on the basis of its influence on TMD.^31^ The procedure was conducted using a Sonopuls 692 device (Enraf Nonius BV, Rotterdam, The Netherlands). The therapist fitted two rubber electrodes (6 × 8 cm) into sponges of equal size, previously dampened with warm water. Participants remained seated in an ergonomic chair, and the therapist provided a massage with the sponges following the sequence (Figure 2): (A) superficial stroke over the neck-shoulder for 30-45 seconds; deep sliding movements, by themselves (B) or combined with shoulder drop (C), for 4-5 minutes; (D) bilateral kneading of the upper trapezius (4-5 minutes); (E) slight stretching of cervical muscles (upper trapezius, sternocleidomastoid, and levator scapulae); and repetition of step (A). We used a current bipolar mode, with a carrier frequency of 4000 Hz, an amplitude-modulated frequency of 100 Hz, and the intensity was set to provide a strong and comfortable tingling, without causing muscle twitches. Participants were informed about the possibility of perceived discomfort and had to report it in order to avoid adverse events. The electro-massage protocol lasted 15 minutes^23^ and is available in the supplementary material.

### Outcome measures

Participants attended an initial visit for baseline measurements (before randomization). Then, they began the 2-week intervention protocol and were evaluated immediately after the last treatment session. A follow-up assessment was conducted at 4 weeks during a separate visit.

### Primary outcomes

The primary outcomes were pain-related measures and vertical mouth opening.

A Visual Analogue Scale (0 to 10 cm) to evaluate the current self-reported pain intensity after bilateral palpation of the central myofascial trigger point of the masseter muscles. This scale is one of the most useful tools for pain screening in patients with TMD.^32^ For individuals with chronic pain, a 30% decrease in pain intensity is considered as clinically relevant.^33^ For women with TMD, the minimal clinically important difference (MCID) has been set at 1.9 cm.^34^

Pressure pain thresholds (i.e., the minimum necessary pressure to cause pain) were measured bilaterally with a digital algometer, model FPX 25 (Wagner Instruments, Greenwich, CT, USA) over: a) the masseter muscle, at a site located 1 cm superior and 2 cm anterior from the mandibular angle; and b) the middle point of the upper trapezius muscle belly.^35^ The mean of three consecutive measurements, with a 30-second rest, was used for analysis. Pressure algometry shows acceptable reliability for masticatory structures,^36^ with a MCID of 0.2 kg/cm2 for the masticatory muscles,^34^ and a minimal detectable change ranging between 0.45 to 1.13 kg/cm2 for the upper trapezius.^37^

The maximum pain-free vertical mouth opening (VMO) was recorded with a digital caliper (Schieblehre digital 59112 Fino, Bad Bocklet, Germany). While in supine position, with the head in neutral position, participants were asked to open their mouth as wide as possible without pain. Then, the distance between the upper and lower central incisors was measured. The mean of three measurements was used for analysis. This procedure exhibits good intra- and inter-rater reliability.^38^ The MCID for VMO has been established to be between 6 mm and 9 mm.^39^

### Secondary outcomes

To assess the active cervical ROM, we employed a universal goniometer (Enraf-Nonius BV, Rotterdam, The Netherlands), which is a low-cost, easy-to-use, and highly reliable tool.^40^ Participants remained seated, and measurements were taken three times for each direction, following the sequence: flexion, extension, right and left side bending, and right and left rotation (40). In patients with neck pain, minimal detectable change (MDC) has been observed to range from 5.9º (right side bending) to 9.6º (flexion).^41^ The arithmetic sum of all movements was calculated and defined as overall cervical ROM.^42^

### Sample size calculation

The G*Power software, version 3.1.9.7 (Heinrich-Heine University, Düsseldorf, Germany) was used to estimate the sample size considering a 30% decrease over time in selfreported pain intensity, as the MCID for patients with chronic pain.^33^ We considered two groups and three measurements and assumed a 1:1 distribution ratio of participants in the study groups, an alpha of 0.05, an 80% statistical power, and a medium effect size (η2 ≈ 0.06). This generated a sample of 42 individuals, including an estimated 15% dropout rate, to complete the trial.

### Data analysis

The software IBM Statistics Package for Social Science^®^, v.26 (IBM Corp, NY, USA) was used to perform the statistical processing of data, with an intention-to-treat analysis. The normal distribution of the variables was assessed with the Shapiro-Wilk test. Data are reported as mean ± standard deviation, mean (95% confidence interval, CI), or in absolute numbers (frequency percentages). We used a repeated-measures analysis of variance (ANOVA) to investigate the changes in the outcome measures after intervention, with group (MT or ES therapy plus MT) as the between-subjects factor, and time (pre, post, 4-week) as the within-subjects factor. The estimated effect size was reported with the partial eta squared (small, 0.01≤ η2 ≤ 0.06; medium, 0.06 ≤ η2 ≤ 0.14; or large, η2 > 0.14). For all tests, statistical significance was set at a p< 0.05.

## Results

**Figure 3:**
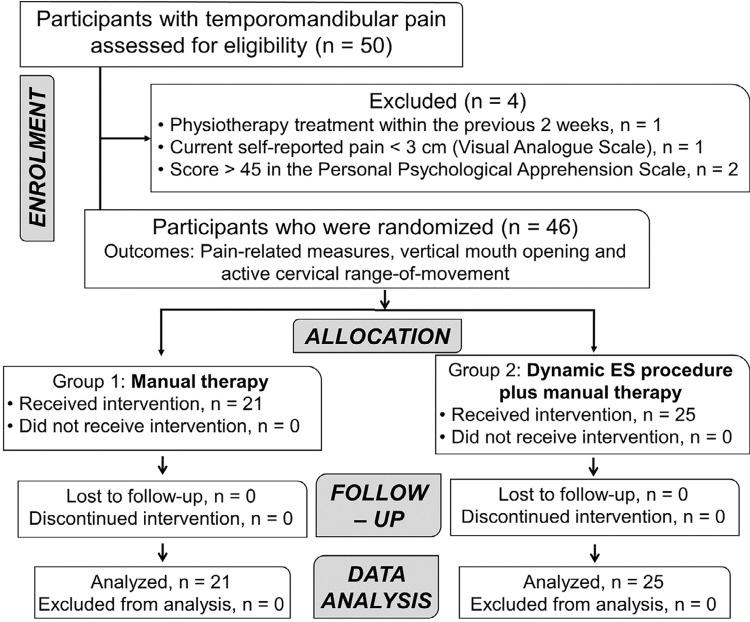
Flowchart diagram of participants

The study included 46 participants with bilateral TMD (80.4% females) who completed the protocol intervention and follow-up assessments, with no adverse events or dropouts reported during the trial (Figure 3). There were no significant differences between groups for baseline clinical data (Table 1).

**Table 1:** Descriptive clinical and demographic features of participants.

Measures	MT group (n = 21)	ES + MT group (n = 25)	P
	Mean (SD)	Mean (SD)	
Mean age, years	26.24 (9.42)	23.92 (7.14)	0.097
Sex: female, n (%)	16 (76.2%)	21 (84%)	0.711
BMI, kg/cm2	23.88 (3.88)	22.88 (2.50)	0.589
PPAS	30 (8.11)	26 (7.07)	0.052

Values are presented ± as mean or number (frequency percentages)

Abbreviations: BMI, body mass index; ES, electrical stimulation; MT, manual therapy; PPAS, Personal Psychological Apprehension Scale.

### Primary outcomes

The ANOVA revealed significant time*group interactions for: (a) self-reported pain intensity (F=15.349; *P*<0.001; η2=0.259); and (b) pressure pain sensitivity at the right masseter muscle (F=14.765; *P*<0.001; η2=0.251); and the upper trapezius (right upper trapezius: F=12.934; *P*<0.001; η2=0.227; left upper trapezius: F=12.558; *P*<0.001; η2=0.222). Similarly, a significant time*group interaction was observed for vertical mouth opening (F=18.858; *P*<0.001; η2=0.300). Participants receiving dynamic ES plus MT exhibited a greater improvement in pain related outcomes and mouth opening, with a large effect size, than those receiving MT by itself (Table 2).

**Table 2:** Pain intensity, pressure pain sensitivity and mouth opening values at baseline (pre), immediately post intervention (post) and at 4-week follow-up (4 week), and within-group and between-groups mean differences.

Outcome (measure)/Group	Preꝉ	Postꝉ	Follow-upꝉ	Within-group scores changesǂ	Between-groups changesǂ
Pain intensity (0 to 10 cm)					
MT	4.90 (1.37)	3.14 (1.35)	3.66 (1.95)	a. 1.76 (1.33 to 2.19); b. 1.23 (0.50 to 1.97)	a. -1.59 (-2.28 to -0.91)*
ES + MT	4.88 (1.12)	1.52 (1.06)	1.74 (1.06)	a. 3.36 (2.79 to 3.92); b. 3.14 (2.64 to 3.63)	b. -1.90 (-2.73 to -1.06)*
PPT (kg/cm2) right masseter muscle					
MT	1.18 (0.14)	1.27 (0.11)	1.17 (0.14)	a. -0.09 (-0.12 to -0.06); b. 0.01 (-0.01 to 0.02)	a. 0.12 (0.02 to 0.21) *
ES+ MT	1.20 (0.28)	1.42 (0.34)	1.46 (0.42)	a. -0.22 (-0.30 to -0.13); b. -0.25 (-0.35 to -0.15)	b. 0.25 (0.15 to 0.36)*
PPT left masseter muscle					
MT	1.16 (0.14)	1.27 (0.13)	1.18 (0.10)	a. -0.11 (-0.15 to -0.07); b. -0.01 (-0.07 to 0.03)	a. 0.08 (0.01 to 0.16)*
ES + MT	1.12 (0.12)	1.33 (0.23)	1.27 (0.30)	a. -0.20 (-0.26 to -0.13); b. -0.14 (-0.26 to -0.02)	b. 0.12 (-0.01 to 0.25)
PPT right upper trapezius muscle					
MT	1.28 (0.14)	1.32 (0.13)	1.29 (0.11)	a. -0.04 (-0.07 to -0.01); b. -0.01 (-0.04 to 0.02)	a. 0.54 (0.23 to 0.84)*
ES + MT	1.38 (0.23)	1.97 (0.85)	2.10 (0.87)	a. -0.58 (-0.88 to -0.27); b. -0.71 (-1.04 to -0.38)	b. 0.70 (0.37 to 1.03)*
PPT left upper trapezius muscle					
MT	1.28 (0.15)	1.30 (0.15)	1.30 (0.17)	a. -0.01 (-0.03 to -0.01); b. -0.01 (-0.04 to 0.01)	a. 0.49 (0.16 to 0.82)*
ES + MT	1.32 (0.33)	1.84 (0.82)	2.08 (0.82)	a. -0.51 (-0.84 to -0.18); b. -0.75 (-1.08 to -0.42)	b. 0.73 (0.40 to 1.06)*
Pain-free vertical mouth opening (mm)					
MT	31.28 (3.90)	33 (3.60)	31.64 (3.46)	a. -1.71 (-2.14 to -1.27); b. -0.35 (-0.94 to 0.22)	a. 7.36 (4.87 to 9.86)*
ES + MT	31.76 (3.13)	40.84 (6.38)	38.16 (6.86)	a. -9.08 (-11.54 to -6.61); b. -6.40 (-9.07 to -3.72)	b. 6.04 (3.31 to 8.77)*

ꝉ Values are mean ± standard deviation.

ǂ Values in parentheses are 95% confidence interval.

* Statistical significance between groups (P < 0.05).

a. Pre-post changes; b. Pre-follow up changes.

Abbreviations: ES, electrical stimulation; MT, manual therapy; PPT, pressure pain threshold

### Secondary outcomes

The ANOVA showed significant time*group interactions (with moderate to large effect sizes) for active cervical ROM in all directions, except for neck rotation: (a) flexion, F=12.024; *P*<0.001; η2=0.215; (b) extension, F=6.858; *P*=0.003; η2=0.135; (c) right side bending, F=24.387; *P*<0.001; η2=0.357; (d) left side bending, F=21.775; *P*<0.001; η2=0.331; (e) right rotation, F=0.885; *P*=0.38; η2=0.020; (f) left rotation, F=1.607; *P*=0.21; η2=0.035; and (g) overall ROM: F=14.382; *P*<0.001; η2=0.246). Individuals in the group 2 (dynamic ES) demonstrated a greater improvement in cervical mobility, compared to those in the group 1 (MT by itself) (Table 3).

**Table 3:** Active cervical range-of-motion values at baseline (pre), immediately post intervention (post) and at 4-week follow-up (4 week), and within-group and between-groups mean differences.

Outcome (measure)/Group	Preꝉ	Postꝉ	Follow-upꝉ	Within-group scores changesǂ	Between-groups changesǂ
Flexion (degrees)					
MT	37.33 (3.18)	37 (4.09)	36.61 (4.06)	a. 0.33 (-1.01 to 1.67); b. 0.71 (-0.59 to 2.01)	a. 4.73 (2.35 to 7.10)*
ES + MT	45.52 (6.49)	49.16 (6.63)	48.34 (6.83)	a. -4.40 (-6.32 to -2.47); b. -4.10 (-6.43 to -1.76)	b. 4.81 (2.19 to 7.43)*
Extension					
MT	45.14 (4.22)	46.19 (4.14)	45.42 (3.47)	a. -1.04 (-1.84 to -0.25); b. -0.28 (-1.03 to 0.46)	a. 2.59 (1.04 to 4.14)*
ES + MT	45.52 (6.49)	49.16 (6.63)	48.34 (6.83)	a. -3.64 (-5.01 to -2.27); b. -2.82 (-4.43 to -1.20)	b. 2.53 (0.78 to 4.28)*
Right side bending					
MT	31.33 (2.92)	30.14 (3.85)	29.69 (4.36)	a. 1.19 (-0.15 to 2.53); b. 1.64 (0.21 to 3.07)	a. 7.71 (5.27 to 10.14)*
ES + MT	34.12 (6.11)	40.64 (8.22)	38.30 (9.38)	a. -6.52 (-8.51 to -4.52); b. -4.18 (-6.59 to -1.76)	b. 5.82 (2.95 to 8.69)*
Left side bending					
MT	30.28 (2.55)	29.76 (3.12)	29.40 (3.42)	a. 0.52 (-0.92 to 1.97); b. 0.88 (-0.48 to 2.24)	a. 7.56 (4.95 to 10.17)*
ES + MT	32.84 (7.52)	39.88 (9.64)	37.72 (9.37)	a. -7.04 (-9.16 to -4.91); b. -4.88 (-7.28 to -2.47)	b. 5.76 (2.92 to 8.59)*
Right rotation					
MT	44.85 (3.49)	47.04 (3.20)	46.61 (3.15)	a. -2.19 (-3.09 to -1.28); b. -1.76 (-2.48 to -1.04)	a. 1.52 (-0.78 to 3.84)
ES + MT	43.80 (2.97)	47.52 (5.81)	46.44 (6.15)	a. -3.72 (-5.88 to -1.55); b. -2.64 (-5.23 to -0.04)	b. 0.87 (-1.79 to 3.54)
Left rotation					
MT	45.23 (3.25)	48.09 (4.12)	45.95 (4.34)	a. -2.85 (-4.25 to -1.45); b. -0.71 (-2.21 to 0.78)	a. 1.14 (-1.44 to 3.72)
ES + MT	44.32 (3.18)	48.32 (5.67)	47.24 (5.56)	a. -4.00 (-6.12 to -1.87); b. -2.92 (-5.33 to -0.50)	b. 2.20 (-0.69 to 5.10)
Overall range-of-movement					
MT	234.19 (15.46)	238.23 (14.52)	233.71 (14.92)	a. -4.04 (-8.02 to -0.06); b. 0.47 (-2.81 to 3.76)	a. 25.27 (15.16 to 35.38)*
ES + MT	240 (22.49)	269.32 (35.79)	261.54 (38.19)	a. -29.32 (-38.77 to -19.86); b. -21.54 (-33.1 to -9.97)	b. 22.01 (10.08 to 33.94)*

ꝉ Values are mean ± standard deviation.

ǂ Values in parentheses are 95% confidence interval.

* Statistical significance between groups (P < 0.05).

a. Pre-post changes; b. Pre-follow up changes.

Abbreviations: ES, electrical stimulation; MT, manual therapy.

## Discussion

The results showed that both conservative interventions improved pain measures and vertical mouth opening. However, the experimental intervention combining dynamic ES and MT was superior to the isolated use of MT to relieve pain and improve mouth opening and neck mobility in people with TMD. These findings may help dentists and orofacial pain specialists in their daily decision-making.

### Pain-related measures

For self-reported pain intensity, the differences between groups surpassed the 30% decrease of the baseline score (≈ 1.47cm), as the clinically meaningful threshold for individuals with chronic pain,^33^ both immediately after intervention (-1.59 cm, 95% CI [2.28 to -0.91] cm) and at the 4-week follow-up (-1.90 cm, 95%CI [-2.73 to -1.06] cm). However, the results after intervention were below the 1.90 cm clinically relevant threshold recently established for women with TMD.^34^ In addition, the combination of dynamic ES plus MT over the neck-shoulder region led to a greater reduction in pain than previous research of ES therapy with TENS,^13,19^ which could be due to the combination of techniques (dynamic ES plus MT) and by the different type of current (TENS versus interferential currents). The intervention studied may enhance the activation of endogenous inhibitory mechanisms and reactive hyperemia in the neck-shoulder region by dynamic ES.^23,24^ In addition, the reduction of muscle spasms around the joint by the spinal reflex mechanism derived from MT could also explain our findings.^15,43^

Regarding PPT, clinically relevant changes in the comparison between-groups^34^ were only observed for the right masseter muscle at the follow-up (0.25 kg/cm2, 95 % CI [0.15 to 0.36] kg/cm^2^). The upper trapezius was also within the minimal detectable change.^37^ Previous research using TENS did not found significant differences in upper trapezius,^19^ which could be explained by the capacity of interferential currents to reach deep structures and to increase blood flow.^21,44,45^

Our results cautiously suggest that a dynamic ES procedure (electro-massage) with IFC delivered distally to the targeted area may achieve an analgesic effect over the masticatory muscles, which could be explained by the relationship between the cervical spine and the TMJ region.^29,46^ More research is needed to understand the analgesic effect of ES therapy with IFC, with and without other approaches, in people with chronic TMD.^45^

### Vertical mouth opening

For pain-free maximum mouth opening, there were significant differences between groups in favour of group 2 (dynamic ES procedure with IFC plus MT), immediately after intervention (7.36 mm, 95%CI [4.87 to 9.86] mm) and at the follow-up (6.4 mm, 95% CI [3.31 to 8.77] mm), which reaches the MCID for this outcome measure.^34^ Our results are similar to previous findings for TENS^13^, as ES with IFC was no better than placebo to increase jaw opening in individuals with recurrent mandibular pain^47^ or knee ROM following arthroplasty.^48^ These controversial findings could be explained by a lack of consensus on the optimal parameters of application of IFC.^21^ In addition, the scarce and heterogenous research assessing the effect of ES modalities on joint ROM makes it difficult to reach a definite conclusion. As the dynamic ES procedure with IFC seems to induce similar improvements in mouth opening to other cervical interventions,^49^ future studies are needed to clarify the clinical effectiveness of a combined application with other cervical techniques versus local manual therapy in patients with myofascial TMD.

### Cervical range of motion

Group 2 reported significant superior improvements for this measure (Table 3). Adding a dynamic ES procedure with IFC to MT led to better results for overall range of movement, with changes between groups ranging from 25.27°, 95% IC (15.16 to 35.38°) after two weeks, to 22.01°, 95% CI (10.08 to 33.94) at the 4 weeks follow-up. However, when considering individual neck movements, differences between groups did not reach the MDC in any direction,^41^ except for right side bending (>5.9º) after two weeks of intervention.

Thus, despite the association between TMD and neck function,^50^ there is scant research on the effect of cervico-mandibular interventions on cervical movement impairments in patients with myofascial temporomandibular pain. Our findings agree with former studies in adults with myofascial pain syndrome, in which using ES therapy with IFC over the neck-shoulder, by itself or with MT, helped to increase active cervical ROM.^51^ Similarly, orofacial treatment in addition to cervical MT has shown to be more effective than cervical MT by itself to enhance neck ROM in patients with signs of TMD,^52^ whereas combining MT and exercise therapy over the neck can increase both cervical ROM and TMJ function.^53^ The improvements in cervical ROM could be explained by the decrease in the hiperactivity of the head and neck musculature.^31^ However, all these previous trials included participants with different pathological conditions, such as stress, rest or inflammation at the time of assesment, which limits the discussion of our results.

### Strengths and limitations

The relevance of the study findings should be interpreted considering some methodological strengths and limitations. Despite the adequacy of the sample size for statistical power and study purposes, the present findings are mostly applicable to young female adults. It is worth mentioning that the sex distribution of the study sample (80.43% female) is consistent with the general prevalence of TMD, in which women show a two times greater risk of developing TMDs compared to men.^54^ However, despite the fact that no age limit was established, most participants were young adults (18 to 35 years); thus, broader diversity of the sample could have improved the generalizability of the findings. We included, for the first time, patients with diagnosed chronic myofascial temporomandibular pain that received a multimodal intervention including MT plus a dynamic ES procedure (interferential currents electro-massage). Moreover, we ensured concealed allocation, assessor blinding, intention-to-treat analysis, and a follow-up.

This study lacked a sham group to identify a potential placebo effect, and a wait-list group to reflect the natural course of the condition. The treatment protocol was also limited to a 2-week period. However, a similar methodology has been followed previously.^19^ Finally, blinding of participants was not possible, and the duration of treatment sessions slightly differed between groups, which could be a source of bias. In addition, we did not include any oral health-related quality of life assessment or assess pain perception with a more comprehensive tool, such as the McGill Pain Questionnaire^55^ which would have been desirable.

## Conclusion

Combining dynamic ES interferential currents with manual therapy over the neck-shoulder region resulted in significantly better results for pain intensity, pressure pain thresholds, mouth opening, and active cervical ROM than manual therapy by itself in subjects with myofascial temporomandibular pain.
